# Itaconate‐Related Gene Signatures as Prognostic Markers in Colon Cancer: Insights From Transcriptomic and Spatial Analysis

**DOI:** 10.1155/humu/7928082

**Published:** 2026-01-10

**Authors:** Tingting Zhang, Jianchao Meng, Qingyun Wang, Peng Zhang, Hui Li, Hailang Wei, Denggang Chen, Chen Bai

**Affiliations:** ^1^ Department of Clinical Oncology, Taihe Hospital, Hubei University of Medicine, Shiyan, China, hbmu.edu.cn; ^2^ Otorhinolaryngology Department, Taihe Hospital, Hubei University of Medicine, Shiyan City, China, hbmu.edu.cn; ^3^ Department of General Surgery, Taihe Hospital, Hubei University of Medicine, Shiyan, China, hbmu.edu.cn

**Keywords:** colon cancer, Hallmark pathway, itaconate, prognostic model, single-cell transcriptomics, spatial transcriptomics

## Abstract

Colon cancer is one of the most prevalent malignant tumors. Accurate evaluation of patient prognosis and optimization of treatment strategies continue to be major research focuses in colon cancer. Based on The Cancer Genome Atlas (TCGA) database, this study is the first to comprehensively analyze the expression, biological roles, and prognosis of itaconate and Hallmark pathway–related genes in colon cancer using bulk transcriptomics, single‐cell transcriptomics, and spatial transcriptomics data. Through strict screening in 448 colon cancer patients from TCGA database (training set) and 7 colon cancer prognostic models from the Gene Expression Omnibus (GEO) database (including 1473 cases in the validation set), 10 prognosis‐related genes (TIMP1, FJX1, CD36, CXCL1, ETS2, CDKN2A, INHBB, PLEC, TUBB2, and P4HA1) were selected. The optimal prognostic prediction model (Enet [alpha = 0.2]) was constructed and validated, which showed good prognostic predictive value in both the training and validation sets (average C‐index > 0.7) and was superior to previous conventional clinical features and 22 prognostic models developed by researchers in the past 4 years. ScRNAseq (GSE225857) and spatial transcriptomics analyses clarified the cell‐specific expression and spatial distribution characteristics of these genes in the tumor microenvironment (TME), with high functional scores mainly enriched in epithelial and stromal cells. Tissue microarray (TMA) showed that the high‐risk group had higher tumor mutation burden (TMB) and higher expression of immune checkpoint genes, suggesting higher sensitivity to immunotherapy. Drug sensitivity analysis identified four potentially effective drugs, such as sepantronium bromide, which had better effects on high‐risk patients. This study provides a theoretical basis and new targets for precise prognosis and stratified treatment of colon cancer.

## 1. Introduction

Colon cancer is one of the most prevalent malignant tumors with high incidence and mortality worldwide, and its pathogenesis is affected by multiple factors such as genetic factors, environmental factors, and lifestyle [1, 2]. In recent years, with the advancement of treatment methods and technologies, the early diagnosis rate and cure rate of colon cancer have improved to a certain extent, but the prognosis for advanced patients is still unsatisfactory [3, 4]. It is estimated that there will be 1.93 million new cases of colon cancer and 930,000 deaths in 2020, showing a trend of younger age year by year. Tumor heterogeneity is the main reason for the different prognoses of colon cancer patients [5, 6]. Even patients with the same clinical stage may have great differences in treatment response and survival time [7, 8]. Therefore, finding accurate and reliable biomarkers for prognostic classification and constructing prognostic models have opened up new avenues for individualized treatment of colon cancer [9, 10]. The commonly used prognostic indicators in clinical practice include clinical tumor stage, pathological grade, and presence or absence of lymph node metastasis, but they cannot fully reflect the biological characteristics of tumors [11, 12]. With the application of high‐throughput gene sequencing technology, molecular markers based on gene expression profiles provide a new direction for the prognosis of colon cancer, but their clinical application needs to be verified and improved [13, 14].

As an important metabolic intermediate in the tricarboxylic acid cycle, itaconate is catalyzed by ACOD1 encoded by the IRG1 gene. It was first reported in activated macrophages and has anti‐inflammatory and immune regulatory activities [15, 16]. In recent years, studies have shown that itaconate in the tumor microenvironment (TME) plays an important role in tumor occurrence and development, mainly by regulating metabolic reprogramming, oxidative stress, and immune suppression pathways [17, 18]. Emerging evidence has begun to delineate its multifaceted role in colorectal cancer. For instance, itaconate derivative 4‐octyl itaconate can inhibit aerobic glycolysis by targeting GAPDH to promote a novel form of cell death known as cuproptosis [19]. Moreover, itaconate production in tumor‐associated macrophages has been linked to obesity‐related pathways and patient survival, suggesting a particular immunotherapeutic role [20]. Additionally, itaconate has been shown to enhance oncolytic virotherapy efficacy in resistant colon cancer models [21]. However, the prognostic value of itaconate‐related genes and their interplay with broader oncogenic pathways in colon cancer remain incompletely elucidated.

The Hallmark pathway is composed of a set of gene sets involved in various important biological processes such as cell cycle and immune response. Their abnormal upregulation or inhibition is related to tumor development [22, 23]. Some studies have shown that prognostic models based on genes related to the Hallmark pathway are of great value in various tumors. Abnormal activation of the cell cycle pathway leads to unlimited cell proliferation, and dysregulation of the immune response pathway affects the sensitivity of tumors to immunotherapy [24, 25]. The cross‐role and prognostic value of itaconate‐related genes and Hallmark pathway genes in colon cancer have not been fully clarified, and their cross‐regulation may provide a new perspective for the evaluation of colon cancer prognosis [26, 27].

With the application of high‐throughput sequencing technology, tumor research has gradually entered the era of multiomics. The integration of bulk transcriptomics, single‐cell transcriptomics, and spatial transcriptomics has become an important tool for analyzing colon cancer heterogeneity and microenvironment [28, 29]. Bulk transcriptome analysis can comprehensively reveal the overall gene expression pattern of tumor tissues, which is a commonly used strategy for screening prognostic markers and constructing models, but this averaging effect may miss the heterogeneity between different cells. Single‐cell transcriptomics can reveal cell type composition and gene expression differences at the single‐cell level and can identify important cell subsets and cell functional states in the TME, such as the phenotype and functional variation of tumor‐infiltrating immune cells [30, 31]. Spatial transcriptomics can retain the spatial location information of gene expression, analyze the regional characteristics of gene expression in tumor tissues, and reveal the spatial heterogeneity of the TME [32, 33]. The combination of these three technical methods can comprehensively analyze the biological characteristics of colon cancer from the overall to the single cell and from the plane to the space, thus providing more research information for in‐depth understanding of the pathological mechanism of tumor occurrence and screening of prognostic markers and therapeutic targets [34, 35].

In this study, we systematically analyzed the prognostic roles and spatial distribution of itaconate‐related Hallmark pathway genes in colon cancer using integrated bulk, single‐cell, and spatial transcriptomic data. The objective of our work is to construct and validate a multiomics prognostic model, identify key gene signatures, and explore their potential functional mechanisms and therapeutic implications, thereby providing a theoretical basis for individualized prognosis prediction and treatment strategies in colon cancer.

## 2. Materials and Methods

### 2.1. Data Sources and Preprocessing

The majority of datasets analyzed in this study were obtained from public repositories, specifically TCGA (UCSC Xena), GEO (Home–GEO DataSets–NCBI), and The Cancer Imaging Archive (TCIA) (Welcome to TCIA–TCIA). TCGA‐COAD dataset, which includes RNA sequencing profiles and matched clinical information for 448 patients with colon cancer, was designated as the training cohort. To ensure data consistency, raw counts were transformed into TPM format, followed by log_2_ transformation to minimize systematic bias. For external validation, seven GEO microarray datasets were incorporated, namely, GSE12945 (*n* = 62), GSE17536 (*n* = 177), GSE17537 (*n* = 55), GSE38832 (*n* = 122), GSE39582 (*n* = 579), GSE41258 (*n* = 182), and GSE87211 (*n* = 196) [36–41]. In addition, three GEO datasets (GSE110224, GSE22598, and GSE41328) containing paired tumor and adjacent normal samples were utilized for differential expression analysis [42–44]. GEO microarray data underwent background correction and quantile normalization using the normalizeBetweenArrays function in the limma R package, followed by batch effect removal via the Combat algorithm in the sva package to ensure comparability across datasets. Single‐cell RNA sequencing and spatial transcriptomics data were retrieved from GSE225857 [45], encompassing single‐cell profiles from 11 tumor specimens (originating from 6 patients) and spatial transcriptomic data from 4 primary tumor samples. Single‐cell data preprocessing was conducted in the Seurat package, applying stringent quality control thresholds: mitochondrial gene fraction below 20%, erythrocyte gene fraction below 3%, UMI counts ranging from 200 to 20,000, and detected gene numbers between 200 and 5000. Normalization, identification of highly variable genes, data scaling, and cell cycle regression were then performed. Spatial transcriptomics data were processed using SpaceRanger software, and expression values were normalized and standardized with the SCTtransform method. Furthermore, somatic SNV mutation profiles were acquired from TCGA database, melanoma immunotherapy datasets were downloaded from GEO, and immune‐related data, including immune checkpoint expression and immune infiltration scores, were obtained from the TIDE platform and TCIA database to support subsequent analyses.

### 2.2. Screening of Prognostic Genes and Molecular Typing

To identify prognostic genes, we adopted a multistep strategy. First, the Pearson correlation analysis was performed between the expression of IRG1 and all genes in the Hallmark pathway gene set, and those with an absolute correlation coefficient greater than 0.3 and a *p* value less than 0.05 were retained, resulting in 994 candidate genes. Second, differential expression analysis between tumor and adjacent normal samples was conducted using three datasets with the limma R package. Genes meeting the criteria of adjusted *p* < 0.05 and |FoldChange| > 2 in at least one dataset were considered significant, yielding 326 genes. Third, univariate Cox proportional hazards regression analysis was performed independently on TCGA‐COAD training set and the merged GEO validation cohort, and genes with statistically significant prognostic value (*p* < 0.05) in both cohorts were selected. This integrated approach ultimately identified 10 prognosis‐associated genes: TIMP1, FJX1, CD36, CXCL1, ETS2, CDKN2A, INHBB, PLEC, TUBB2, and P4HA1. Based on these genes, consensus clustering was conducted on TCGA‐COAD cohort using the NMF package, with the optimal number of clusters determined by the cophenetic correlation coefficient. Four distinct molecular subtypes were defined, and their clinicopathological characteristics, including age, sex, and tumor stage, were compared, followed by the Kaplan–Meier survival analysis to assess prognostic differences among subtypes.

### 2.3. Construction and Evaluation of Prognostic Models

Using the 10 identified prognostic genes, we constructed multiple prognostic models by integrating 10 machine learning algorithms into 101 unique combinations. Model parameters were optimized via leave‐one‐out cross‐validation (LOOCV) in TCGA training set. Specifically, for the elastic net model, we performed a grid search to tune the hyperparameter *α* (mixing penalty) across a range of values from 0 to 1 in increments of 0.1, alongside the regularization parameter *λ*. The combination of *α* = 0.2 and the optimal *λ* yielded the highest mean cross‐validated C‐index in the training set and was therefore selected for the final model. Predictive performance was then evaluated by calculating Harrell’s concordance index (C‐index) in the seven GEO validation cohorts. The model with the highest mean C‐index, an elastic net regression model with *α* = 0.2, was selected as the optimal predictive tool. Time‐dependent ROC analysis was used to determine the AUC values for predicting 1‐, 3‐, and 5‐year overall survival, and the Kaplan–Meier survival curves were generated to compare survival outcomes between high‐ and low‐risk groups defined by the model. To benchmark our model against existing approaches, we compared its predictive accuracy to 22 recently published colon cancer prognostic models across eight independent datasets (TCGA plus seven GEO cohorts). In addition, univariate and multivariate Cox regression analyses incorporating risk scores and clinical characteristics were performed to identify independent prognostic factors. A nomogram integrating these factors was developed, and its predictive accuracy and clinical utility were evaluated using calibration plots and decision curve analysis (DCA).

### 2.4. Single‐Cell, Spatial Transcriptomics, and TME Analysis

For single‐cell analysis, the Seurat package (v4.3.0) was used to annotate cell populations based on canonical marker genes, classifying them into epithelial cells, fibroblasts, endothelial cells, immune cells, and other stromal cell types. The AUCell package (v1.16.0) was then employed to calculate functional scores for the 10 prognostic genes at the single‐cell level, and cells were dichotomized into high‐ and low‐score groups according to the median score. Differentially expressed genes between these groups were subjected to Gene Ontology (GO) and Kyoto Encyclopedia of Genes and Genomes (KEGG) pathway enrichment analyses to explore functional differences. For spatial transcriptomics analysis, cell type composition in each spatial spot was estimated by integrating single‐cell annotation results with the CARD algorithm (v0.1.0). Functional scores and spatial distributions were mapped, and pathway enrichment in high‐score regions was evaluated using Gene Set Enrichment Analysis (GSEA, v4.3.2). The TME landscape was further characterized by estimating immune cell infiltration using the CIBERSORT (v1.06), TIMER (v2.0), MCPcounter (v1.2.0), and ESTIMATE (v1.0.13) algorithms. Immune infiltration patterns were compared between high‐ and low‐risk groups, and the tumor mutation burden (TMB) was calculated using the maftools package (v2.14.0). Correlations between TMB, risk score, and the expression of 28 immune checkpoint genes were analyzed to assess potential responsiveness to immunotherapy. In addition, intercellular communication networks were inferred from single‐cell data using the CellChat package (v1.5.0), with a focus on endothelial–epithelial–fibroblast interactions to elucidate their potential role in tumor progression.

### 2.5. Prediction of Immunotherapy Response and Drug Sensitivity

To evaluate the potential clinical relevance of the prognostic model in immunotherapy, risk scores were calculated for patients in two melanoma immunotherapy cohorts (GSE106128 and GSE91061). The Kaplan–Meier survival analysis was performed to compare outcomes between high‐ and low‐risk groups, and associations between risk scores and treatment response were assessed. The TIDE web tool was applied to predict immune response potential in TCGA patients, and differences in risk scores between predicted responders and nonresponders were analyzed. Immunophenoscore (IPS) data obtained from TCIA database were also compared between risk groups to further estimate potential benefits from immunotherapy. Drug sensitivity analysis was performed using the oncoPredict R package to estimate the half‐maximal inhibitory concentration (IC50) values for commonly used chemotherapeutic agents. Differences in IC50 between high‐ and low‐risk groups were assessed using the Wilcoxon rank‐sum test, and agents predicted to be more effective in the high‐risk group, such as sepantronium bromide, were identified as potential candidates for personalized therapy. All statistical analyses were conducted in R Version 4.3.0, and a *p* value < 0.05 was considered statistically significant.

### 2.6. RNA Extraction and Quantitative Real‐Time PCR (qRT‐PCR)

Total RNA was extracted from cell samples using TRIzol reagent (Invitrogen, Carlsbad, California, United States) following the manufacturer’s instructions. RNA concentration and purity were determined using a NanoDrop 2000 spectrophotometer (Thermo Fisher Scientific, Waltham, Massachusetts, United States), with all samples showing an A260/A280 ratio between 1.8 and 2.0. Complementary DNA (cDNA) was synthesized from 1 *μ*g of total RNA using the PrimeScript RT Reagent Kit (Takara, Japan). qRT‐PCR was performed using SYBR Premix Ex Taq (Takara) on a QuantStudio 5 Real‐Time PCR System (Applied Biosystems, United States) to quantify TIMP1 mRNA levels. The thermal cycling protocol consisted of an initial denaturation at 95°C for 30 s, followed by 40 cycles of 95°C for 5 s and 60°C for 30 s. Relative gene expression was calculated using the 2^−*Δ* *Δ*Ct^ method, with GAPDH serving as the internal control. All reactions were performed in technical triplicate.

### 2.7. Cell Culture

The human colorectal adenocarcinoma cell lines SW620 (female, colon, metastatic site: lymph node, American Type Culture Collection (ATCC) CCL‐227, RRID:CVCL_0547) and COLO201 (male, colon, metastatic site: ascites, ATCC CCL‐224, RRID:CVCL_0210) were obtained from the ATCC (Manassas, Virginia, United States) in 2023. Cells were cultured in Dulbecco’s Modified Eagle Medium (DMEM; Gibco, Grand Island, New York, United States) supplemented with 10% fetal bovine serum (FBS; Gibco) and 1% penicillin–streptomycin (Gibco) at 37°C in a humidified atmosphere containing 5% CO_2_.

Cell line authentication was performed by short tandem repeat (STR) profiling at Beyotime Biotechnology (Shanghai, China) in January 2025, which confirmed a ≥ 90% match to the reference profile of each line. According to the International Cell Line Authentication Committee (ICLAC) database, neither SW620 nor COLO201 has been reported as misidentified or cross‐contaminated.

Both cell lines were routinely tested for mycoplasma contamination using the LookOut Mycoplasma PCR Detection Kit (MP0035, Sigma‐Aldrich, St. Louis, Missouri, United States) and were confirmed to be free of mycoplasma during the described experiments.

### 2.8. Small Interfering RNA (siRNA) Transfection

Two siRNAs specifically targeting TIMP1 (siTIMP1‐1 and siTIMP1‐2) and a scrambled negative control siRNA (siNC) were designed and synthesized by TsingKe Biotechnology. Stock solutions were prepared in nuclease‐free water at a concentration of 10 *μ*M. SW620 and COLO 201 cells were seeded in 6‐well plates at 2 × 10^5^ cells/well and incubated overnight. For transfection, 50 nM siRNA was complexed with 5 *μ*L Lipofectamine 3000 (Invitrogen, L3000015) in Opti‐MEM Reduced Serum Medium (Gibco) and incubated at room temperature for 15 min before being added to the cells. After 6 h, the medium was replaced with complete growth medium. At 48 h posttransfection, total RNA was extracted with TRIzol, and TIMP1 expression levels were assessed by qRT‐PCR and western blotting. Knockdown efficiency was determined by the 2^−*Δ* *Δ*Ct^ method, showing over 70% reduction in TIMP1 mRNA expression compared to siNC (*p* < 0.01, Student’s *t*‐test). All experiments were performed in triplicate.

### 2.9. Western Blotting

Total proteins were extracted from cells using RIPA buffer (Beyotime, China) supplemented with protease inhibitors (Roche). Protein concentrations were measured using the BCA Protein Assay Kit (Pierce, Thermo Fisher Scientific). Equal amounts of protein (30 *μ*g per lane) were separated on 10% SDS‐PAGE gels and transferred to PVDF membranes (Millipore). Membranes were blocked with 5% nonfat milk in TBST for 1 h at room temperature and incubated overnight at 4°C with primary antibodies against TIMP1 (1:1000, Proteintech, 16644‐1‐AP) and *β*‐actin (1:5000, Proteintech, 66009‐1‐Ig). After washing, membranes were incubated with HRP‐conjugated secondary antibodies (1:5000, CST) for 1 h at room temperature. Protein bands were visualized using enhanced chemiluminescence (ECL; Millipore) and quantified using ImageJ software.

### 2.10. Cell Proliferation Assay (Cell Counting Kit‐8 [CCK‐8])

Cell proliferation was assessed using the CCK‐8 (Dojindo, Japan) as previously described [46], with minor modifications. Cells were seeded into 96‐well plates at a density of 3 × 10^3^ cells per well and transfected with siRNAs as described above. At 24, 48, 72, and 96 h posttransfection, 10 *μ*L of CCK‐8 solution was added to each well and incubated for 2 h at 37°C. Absorbance was measured at 450 nm using a Synergy H1 microplate reader (BioTek, United States). Each experimental condition was tested in quintuplicate.

### 2.11. Wound Healing Assay

The effect of TIMP1 silencing on cell migration was evaluated by a wound healing assay. COLO 201 and SW620 cells transfected with siNC, siTIMP1‐1, or siTIMP1‐2 were seeded into 6‐well plates and grown to 90%–100% confluence. A sterile 200‐*μ*L pipette tip was used to create a uniform scratch, and detached cells were removed by washing with PBS. Serum‐free medium was added to minimize proliferation, and images were captured at 0 and 48 h using a phase‐contrast microscope. Each condition was tested in triplicate, and the experiment was repeated independently at least three times.

### 2.12. Migration and Invasion Assays

Cell migration assays were conducted using Transwell chambers (8 *μ*m pore size; Corning, United States). A total of 5 × 10^4^ cells suspended in serum‐free medium were seeded into the upper chamber, and medium containing 10% FBS was placed in the lower chamber as a chemoattractant. After 24 h of incubation at 37°C, cells remaining on the upper membrane surface were removed, and migrated cells on the lower surface were fixed with 4% paraformaldehyde, stained with 0.1% crystal violet, and counted in five randomly selected fields under a microscope. For invasion assays, the same protocol was followed except that the inserts were precoated with Matrigel (BD Biosciences, United States) diluted 1:8 in DMEM and incubated at 37°C for 4 h prior to cell seeding.

## 3. Results

### 3.1. Screening and Characterization of Prognostic Genes

Target gene set description analysis found that IRG1 was significantly correlated with 50 Hallmark pathway gene sets. The pathway correlation heat map intuitively showed the relationship between IRG1 and various pathways, among which pathways such as epithelial–mesenchymal transition and inflammatory response were closely related to IRG1 (Figure 1a). Analysis of TCGA, GSE110224, GSE22598, and GSE41328 datasets found that 994 related genes had significant expression differences between tumor and adjacent samples, and the volcano map showed the fold change and significance of each gene (Figure 1b). The forest plot of univariate Cox regression analysis showed the hazard ratio (HR) and 95% confidence interval of the 10 prognostic genes, among which CDKN2A, TIMP1, and other genes had higher HR, suggesting that they may be factors of poor prognosis (Figure 1c). The heat map of expression correlation of these 10 prognostic genes in each dataset showed that some genes had obvious coexpression trends. CXCL1 was positively correlated with ETS2, and PLEC was negatively correlated with TUBB2, suggesting that these genes may affect tumor development through synergistic effects (Figure 1d). The results showed that the 10 screened prognostic genes had biologically significant differences and prognostic value, laying a foundation for the establishment of subsequent prognostic models.

Figure 1Characterization results of the target gene set. (a) Correlation heat map of the itaconic acid gene (IRG1) and 50 gene sets related to Hallmark pathways. (b) Differential volcano plots of genes related to two gene sets in tumors and adjacent normal tissues from TCGA, GSE110224, GSE22598, and GSE41328. (c) HR forest plot of the combined dataset of TCGA and GEO. (d) Correlation heat map of the expression levels of 10 prognostic genes from TCGA, GSE110224, GSE22598, and GSE41328.

**Figure:**
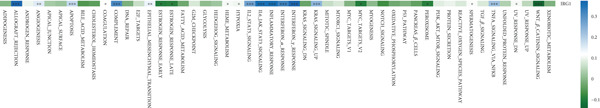
(a)

**Figure:**
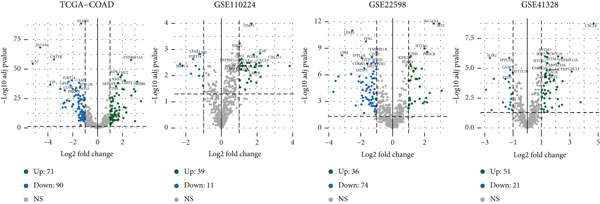
(b)

**Figure:**
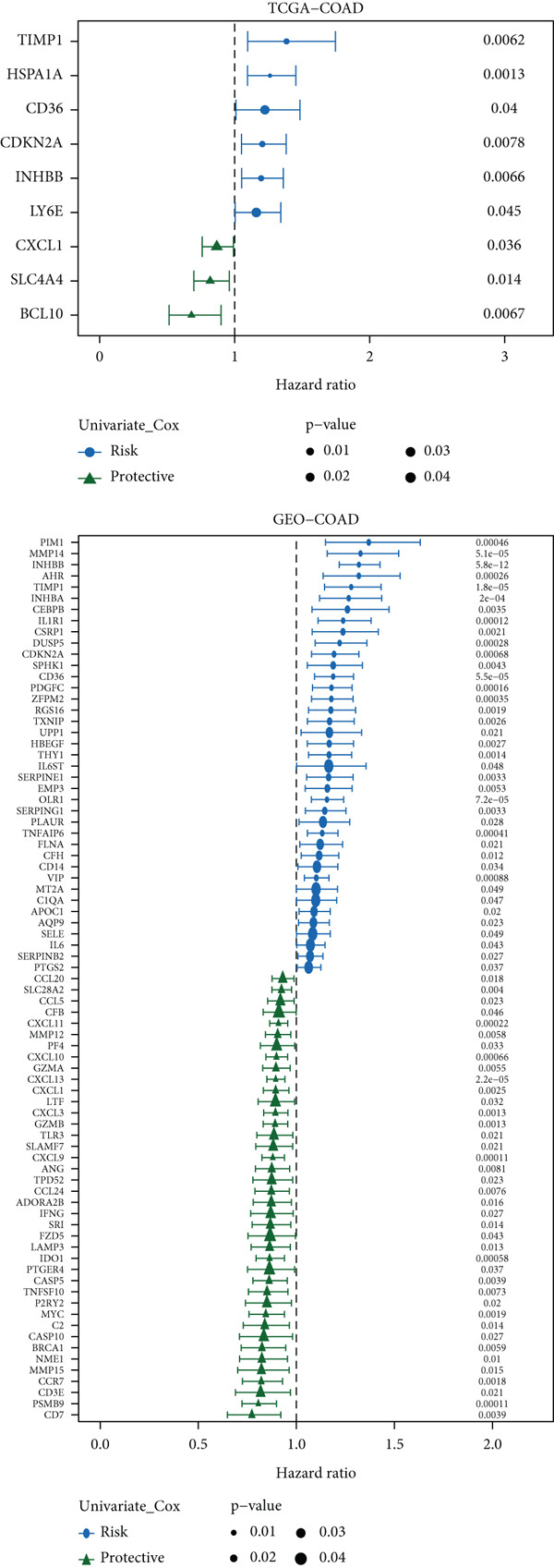
(c)

**Figure:**
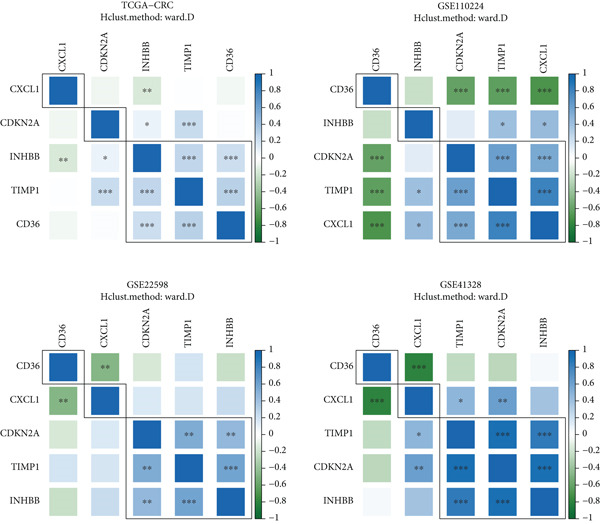
(d)

### 3.2. Association Between Molecular Typing and Clinical Features

Based on NMF clustering analysis of 10 prognostic genes, when divided into 4 subtypes, the cophenetic coefficient was the highest, and the clustering result was optimal (Figure 2a). The consensus clustering heat map reflected different gene expression patterns among the four subtypes (Cluster 1, C1; Cluster 2, C2; Cluster 3, C3; and Cluster 4, C4). Survival analysis found that patients with subtype C1 had the worst prognosis, subtype C3 had the best prognosis, and the survival rate between C1 and C3 subtypes was significantly different (*p* < 0.001) (Figure 2a). Combined clinical feature analysis suggested that the four subtypes had significant differences in age, gender, tumor stage, and pathological grade. For example, subtype C1 had more advanced patients, and subtype C3 had more early‐stage patients (Figure 2b). The Sankey diagram of TCGA immune typing combined with NMF subtypes showed that most of subtype C1 corresponded to immune exclusion type (C2), and most of subtype C3 corresponded to immune inflammation type (C1), indicating significant differences in immune microenvironment among different subtypes (Figure 2c). The volcano map of differential genes between C1 and C3 showed that the upregulated genes were mainly enriched in pathways such as cell cycle and EMT, and the downregulated genes were mainly enriched in immune response pathways (Figure 2d,e). The heat map of the correlation between enriched pathways and ssGSEA scores of 10 prognostic genes showed that the cell cycle pathway was positively correlated with the scores of CDKN2A, TIMP1, and other genes, and the immune pathway was negatively correlated with the scores of CXCL1 and other genes (Figure 2f). The above results indicated that the molecular typing of colon cancer was closely related to clinical features and immune microenvironment, providing scientific support for understanding tumor heterogeneity.

Figure 2Results of functional characterization–molecular subtyping. (a) NMF clustering results, consistency heat map, and survival analysis plot of 10 prognostic genes. (b) Bar chart of NMF classification combined with clinical indicators such as age, gender, stage, and pathological grade. (c) The Sankey diagram of the composition of TCGA immune subtypes, TCGA subtypes, and NMF groups. (d) Volcano plot of gene differences between C1 and C3. (e) GSEA plot of enrichment analysis of upregulated and downregulated genes. (f) Correlation heat map between the enriched pathways and the ssGSEA scores of 10 related prognostic genes.

**Figure:**
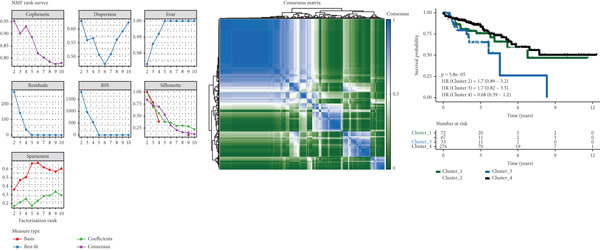
(a)

**Figure:**
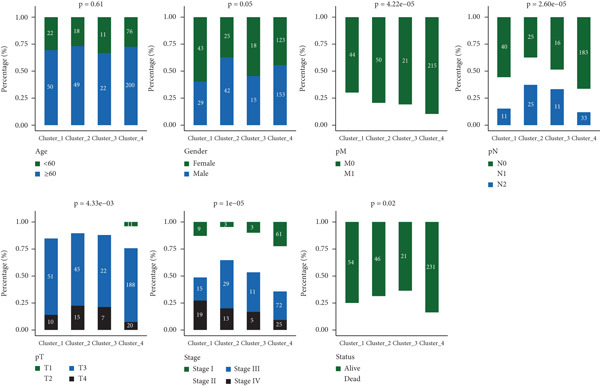
(b)

**Figure:**
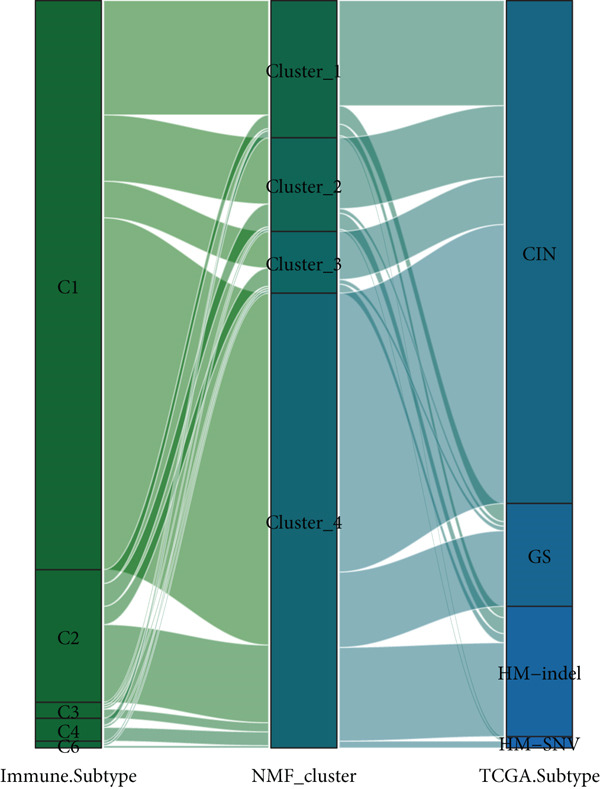
(c)

**Figure:**
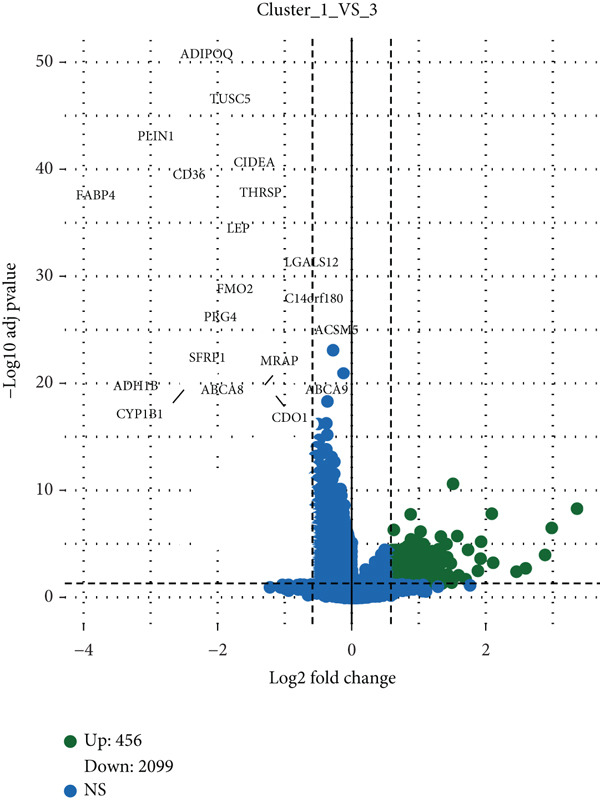
(d)

**Figure:**
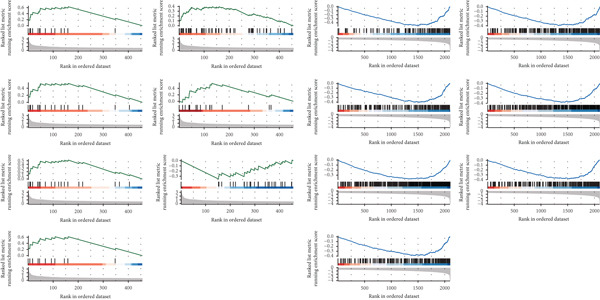
(e)

**Figure:**
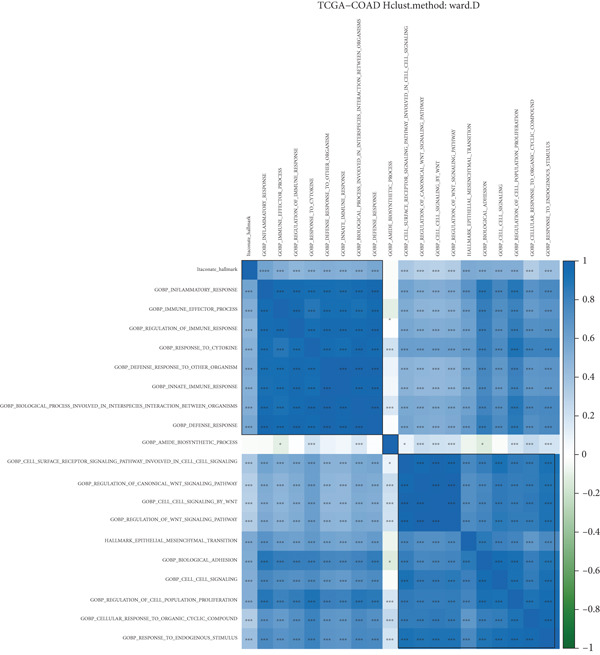
(f)

### 3.3. Single‐Cell and Spatial Transcriptomics Analyze Gene Function Distribution

After single‐cell analysis and cell classification, the functional scores of the 10 prognostic genes also differed among different cell types, with higher functional scores in endothelial cells, fibroblasts, myeloid cells, and epithelial cells (Figure 3a). The enrichment results of differential genes between high and low functional score cells showed that the pathways enriched in high functional score cells mainly included cell proliferation and angiogenesis, and the pathways enriched in low functional score cells mainly included immune response pathways (Figure 3a). Spatial transcriptomics analysis using CRC_C3 as a sample showed that immune cells were mainly distributed in the stromal region, epithelial cells in the parenchymal region, stromal cells evenly distributed, and high functional score regions distributed at the junction of stroma and epithelium (Figure 3b). The functional score was positively correlated with the proportion of epithelial cells and negatively correlated with the proportion of stromal cells (Figure 3c). When the functional score was high, the main pathways enriched by GSEA were cell cycle, DNA repair, and so forth, which was consistent with the results of single‐cell analysis (Figure 3c). The above results indicated the cell specificity and spatiality of the function of prognostic genes, providing new insights for understanding the mechanism of gene function.

Figure 3Functional characterization–single‐cell and spatial transcriptome results. (a) UMAP plots of cell annotation, functional grouping, and functional scores from single‐cell analysis, violin plots of functional analysis, and GSEA plots of functional enrichment for high and low functional scores. (b) H&E staining plots of immune, epithelial, stromal, and functional scores of spatial transcriptomics data. (c) Correlation plots of epithelial and stromal scores with functions in spatial transcriptomics data, curve plots of epithelial, immune, and stromal scores corresponding to spots arranged in ascending order of functional scores and GSEA plots of functional enrichment for the high functional score group.

**Figure:**
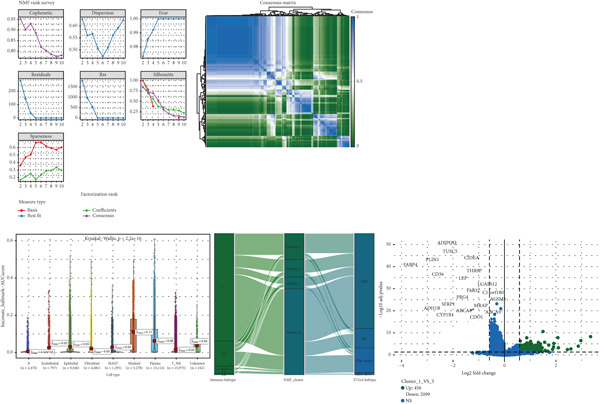
(a)

**Figure:**
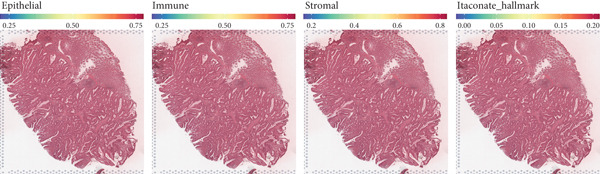
(b)

**Figure:**
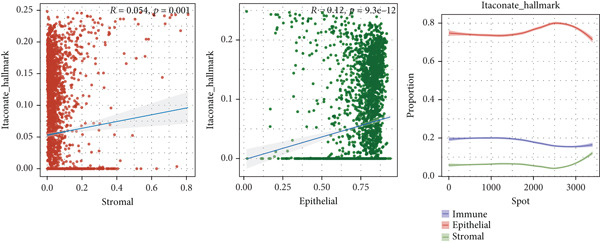
(c)

### 3.4. Construction and Evaluation of Prognostic Models

The C‐index heat map of 101 algorithms in 7 validation sets showed that the optimal model Enet (alpha = 0.2) had the highest average C‐index (0.73), which was determined as the optimal model (Figure 4a). The AUC values at 1, 3, and 5 years in eight datasets were all > 0.65, among which the 5‐year AUC of TCGA dataset reached 0.78, indicating that the constructed model has good predictive performance (Figure 4b). The bar chart of the C‐index of the optimal model in each dataset showed good stability (Figure 4c). Survival analysis results of eight datasets showed that the survival rate of patients in the high‐risk group was significantly lower than that in the low‐risk group (*p* < 0.05), indicating high model reliability (Figure 4d).

Figure 4Construction results of the prognostic model based on differential genes. (a) C‐index heat map of 101 algorithms and 7 validation datasets. (b) AUC values for 1, 3, and 5 years in eight datasets. (c) Bar chart of the C‐index of the optimal model in each dataset. (d) Survival analysis results of eight datasets.

**Figure:**
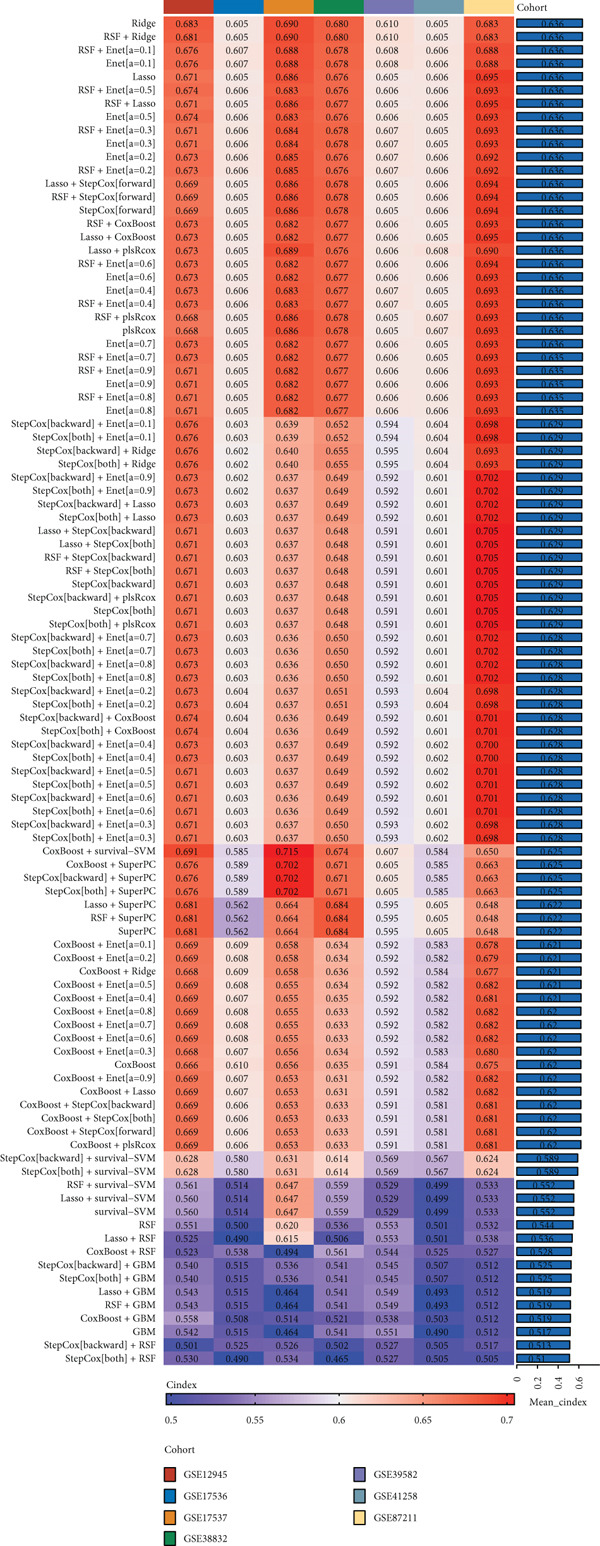
(a)

**Figure:**
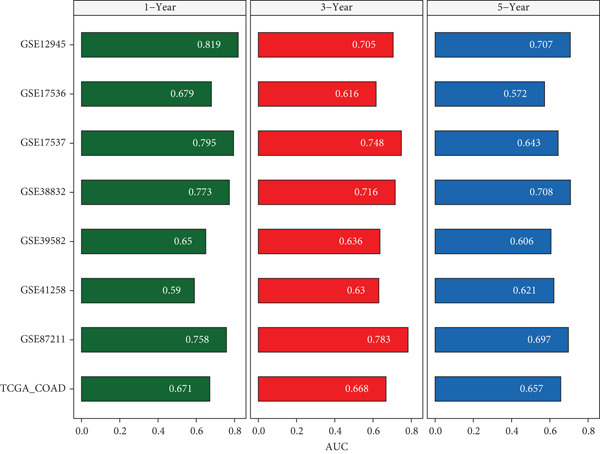
(b)

**Figure:**
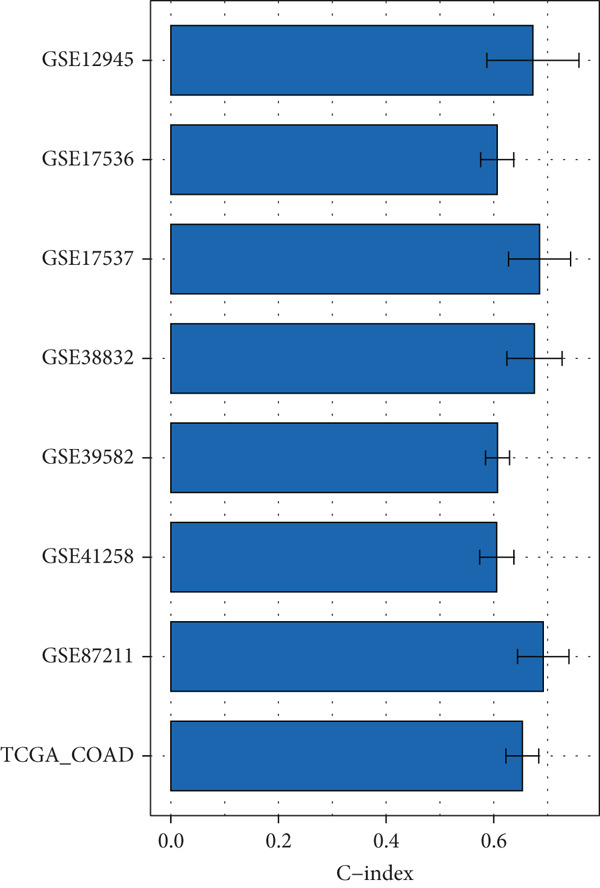
(c)

**Figure:**
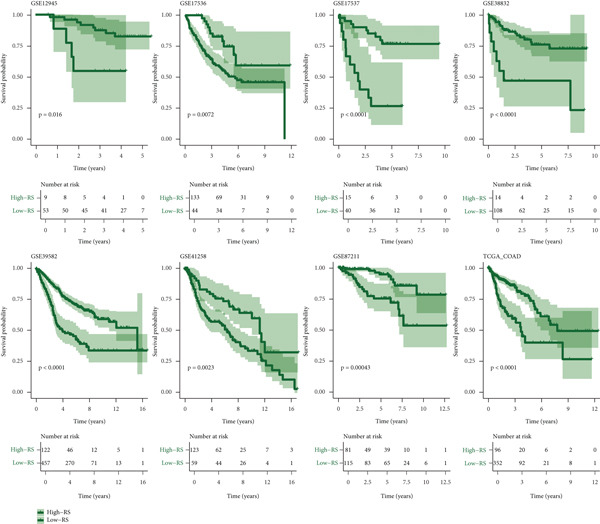
(d)

### 3.5. Prognostic Model Comparison: Superior to Clinical Indicators and Previous Models

The riskplot (Figure 5a) and PCA plot (Figure 5b) of eight datasets showed that high‐ and low‐risk groups could be clearly distinguished in terms of risk score distribution and gene expression patterns. The C‐index of risk score was compared with that of other clinical indicators, and the results were presented as a bar chart (Figure 5c), showing that the C‐index of risk score was better than most clinical indicators. In 8 datasets, the prognostic model of this study was compared with 22 other prognostic models in terms of C‐index, and the results (Figure 5d) showed that this model was generally better than most other models.

Figure 5Comparison results of prognostic models. (a, b) Riskplot and PCA plots of eight datasets. (c) Bar chart of C‐index for risk scores and other clinical indicators. (d) C‐index plot comparing our prognostic model with 22 other prognostic models in 8 datasets.

**Figure:**
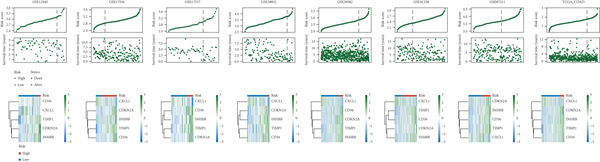
(a)

**Figure:**
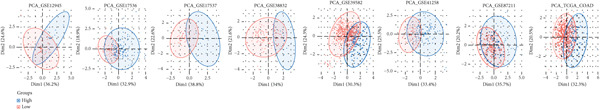
(b)

**Figure:**
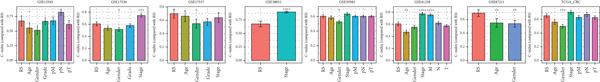
(c)

**Figure:**
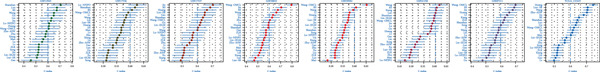
(d)

### 3.6. Nomogram Model: Clinical Application Value

The results of univariate and multivariate analyses of risk score and clinical indicators were presented as a forest plot (Figure 6a), showing that risk score was an independent prognostic factor. The nomogram plot combining risk score and clinical indicators (Figure 6b) could be used to predict the 1‐, 3‐, and 5‐year survival rates of patients. The results of DCA analysis (Figure 6c) showed that the results of nomogram and risk score were better than other clinical indicators. The calibration curves at 1, 3, and 5 years (Figure 6d) showed high consistency between the predicted values of the model and the actual observed values. The results of survival analysis using nomogram score (Figure 6e) showed that patients with high scores had a poor prognosis.

Figure 6Results of the establishment of the nomogram model. (a) Univariate and multivariate analysis results and forest plot of risk scores and clinical indicators. (b) Univariate and multivariate analysis results and forest plot of risk scores and clinical indicators. (c, d) DCA result graph, calibration curves for 1, 3, and 5 years. (e) Result graph of survival analysis using nomogram scores.

**Figure:**
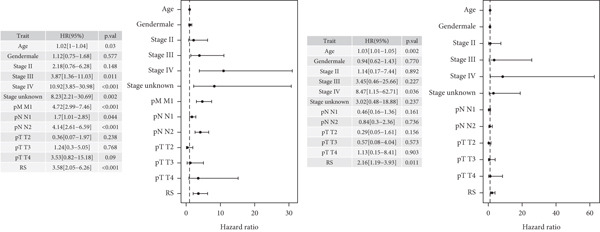
(a)

**Figure:**
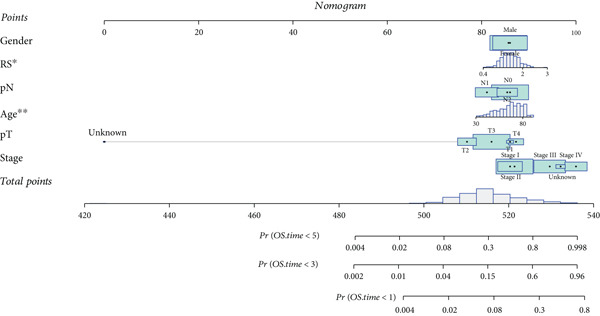
(b)

**Figure:**
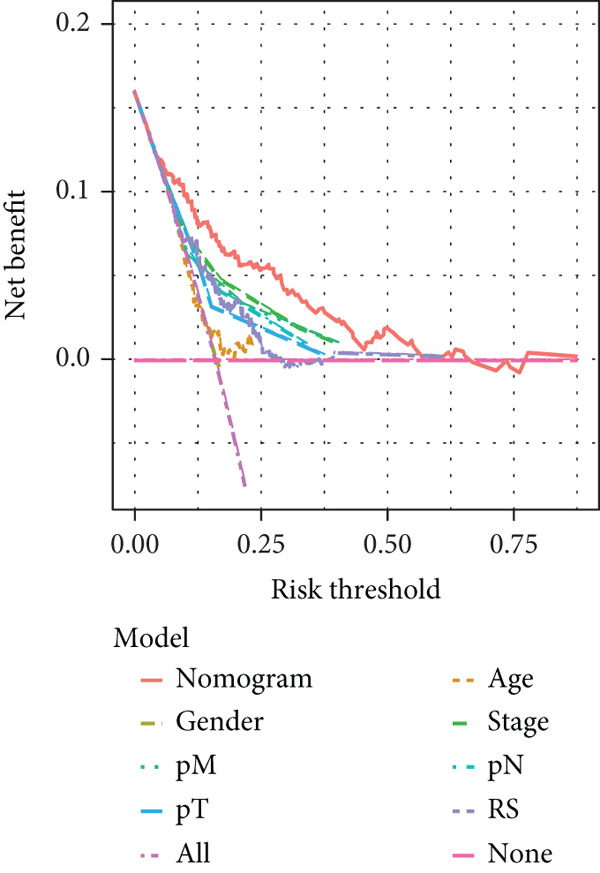
(c)

**Figure:**
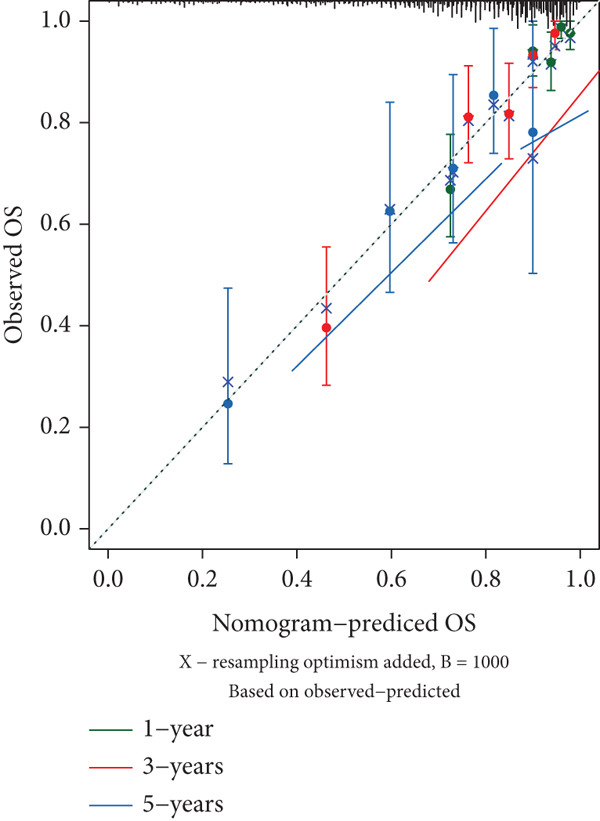
(d)

**Figure:**
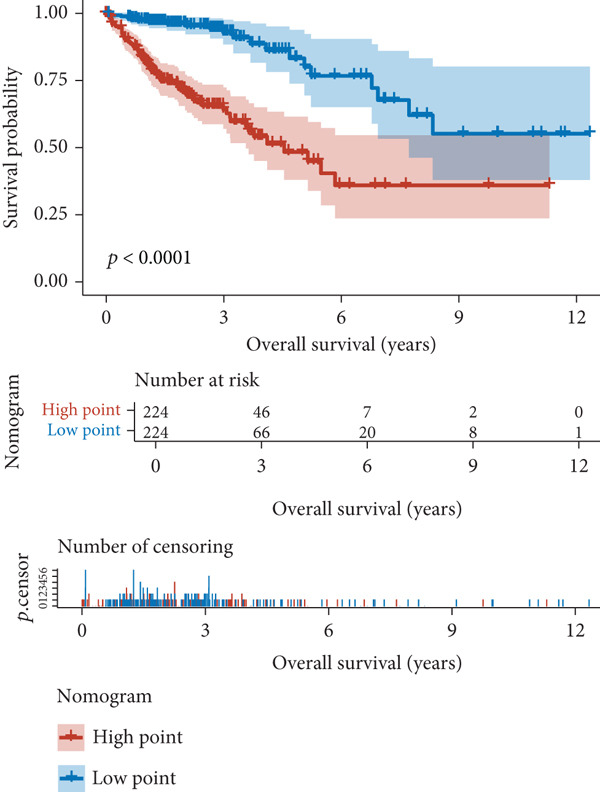
(e)

### 3.7. Tumor Immune Infiltration and TMB Analysis

The box plot of risk values of four NMF classification groups (left of Figure 7a) showed significant differences in risk values among the four groups. The correlation heat map between risk score and 50 Hallmark gene sets (middle of Figure 7a) showed that risk score was significantly correlated with multiple pathways. The box plot of TMB in the high‐ and low‐risk groups (right of Figure 7a) showed that the high‐risk group had higher TMB (*p* < 0.05).

Figure 7Results of tumor immune infiltration analysis and TMB analysis. (a) Box plots of risk values in the four groups of NMF, heat map of the correlation between risk values and 50 Hallmark gene sets, and box plots of TMB in the two risk groups. (b) Box plots of immune, stromal, ESTIMATE scores, and tumor purity in the two groups. The CIBERSORT algorithm shows the box plots of the differences in the infiltration of immune cells in the two groups, and the heat map of the infiltration levels of other immune infiltration algorithms.

**Figure:**
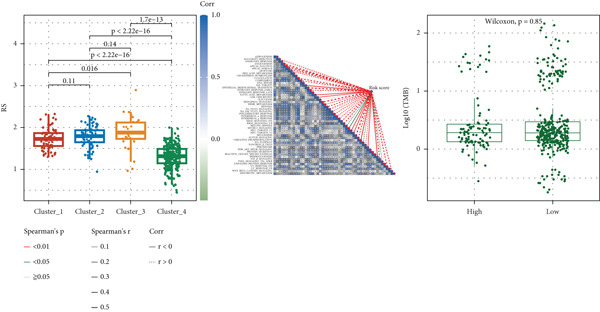
(a)

**Figure:**
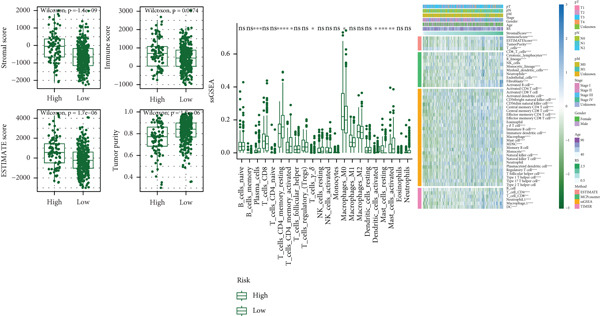
(b)

The box plots of differences in immune, stromal, ESTIMATE scores, and tumor purity between the high‐ and low‐risk groups (upper of Figure 7b) showed significant differences in these indicators between the two groups. The CIBERSORT algorithm was used to analyze the differences in immune cell infiltration between the two groups, and the results were presented as a box plot (middle of Figure 7b), showing that the infiltration degree of some immune cells was significantly different between the two groups. The correlation heat map of infiltration levels from other immune infiltration algorithms (MCP‐counter and TIMER) and risk scores (lower of Figure 7b) further verified the association between immune infiltration and risk scores.

### 3.8. Immunotherapy and Drug Sensitivity Prediction

The correlation heat map between risk score and immune checkpoint genes (left of Figure 8a) showed that risk score was positively correlated with most immune checkpoint genes. The composition bar chart of TIDE (middle of Figure 8a) and the box plot of risk values (middle right of Figure 8a) showed that the composition of responsive and nonresponsive groups in the two risk groups was significantly different, and the nonresponsive group had higher risk scores. The box plot of IPS in the high‐ and low‐risk groups (right of Figure 8a) showed that the low‐risk group had higher IPS scores.

Figure 8Results of immunotherapy analysis and drug sensitivity analysis. (a) Correlation heat map between risk score and immune checkpoints, composition histogram of TIDE, box plot of TIDE risk value, and box plot of IPS. (b) Survival analysis results of immunotherapy cohorts GSE106128 (melanoma) and GSE91061 (melanoma) datasets and risk scores of the two groups of immune responses. (c) Differential box plots of sepantronium bromide, staurosporine, luminespib, and AZD8055 in the high‐ and low‐risk groups.

**Figure:**
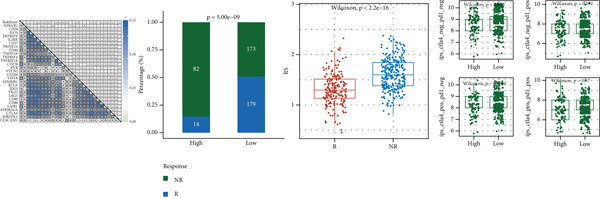
(a)

**Figure:**
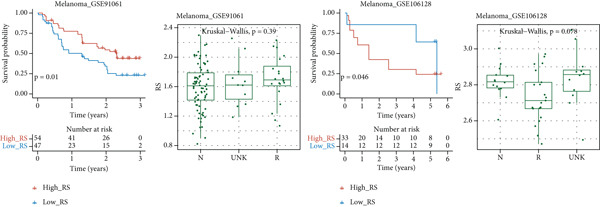
(b)

**Figure:**
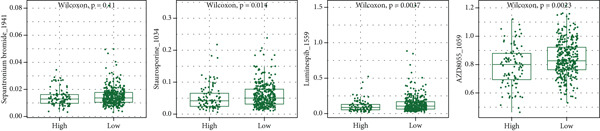
(c)

The survival analysis results of immunotherapy cohorts GSE106128 (melanoma) and GSE91061 (melanoma) datasets (upper of Figure 8b) showed that patients in the low‐risk group had better prognosis. The box plot of risk scores in two immune response groups (lower of Figure 8b) showed that responders had lower risk scores.

The results of drug sensitivity analysis (Figure 8c) showed that the IC50 of sepantronium bromide, staurosporine, luminespib, and AZD8055 was significantly different between the high‐ and low‐risk groups, and the high‐risk group was more sensitive to these drugs.

### 3.9. Differences in Cell Communication at Single‐Cell Level

After calculating and grouping risk values for each cell in the single‐cell dataset, CellChat was used for cell communication analysis. The results showed (Figure 9a) that some communication pathways (such as PCIFI‐POCIFA and MMPT‐(HGF+ITGA)) between endothelial, epithelial, and fibroblasts in the high‐risk group were significantly enhanced. Meanwhile, the results also showed (Figure 9b) that the signals of some communication pathways (such as WNESA‐MCAM and SMAJC‐PLXNDI) in the high‐risk group were significantly weakened. These differences revealed different characteristics of cell communication between high‐ and low‐prognostic‐risk cells.

Figure 9Results of cell communication differences between cells with high and low prognostic risks at the single‐cell level. (a) Heat map of communication differences in endothelial, epithelial, and fibroblast cells between the two groups (increased in the high‐risk group). (b) Bubble plots of communication differences in endothelial, epithelial, and fibroblast cells between the two groups (decreased in the high‐risk group).

**Figure:**
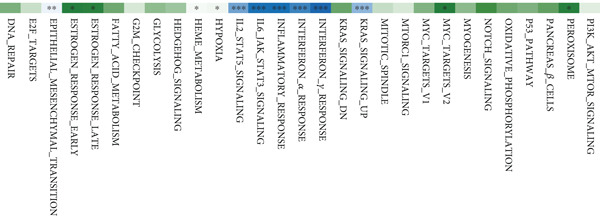
(a)

**Figure:**
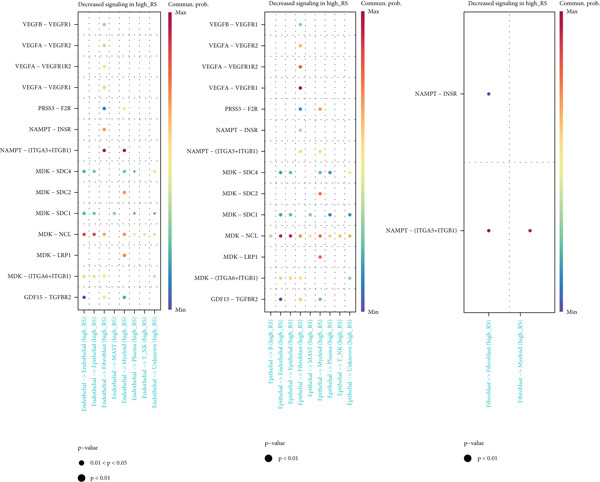
(b)

### 3.10. Functional Validation of TIMP1 Expression and Its Oncogenic Role in CRC

To validate the functional relevance of TIMP1 in CRC, we investigated the functional role of TIMP1 in CRC cell lines by transfecting SW620 and COLO 201 cells with two independent siRNAs (siTIMP1‐1 and siTIMP1‐2). qRT‐PCR analysis confirmed a marked reduction in TIMP1 mRNA levels in both cell lines after transfection (Figure 10a), and western blot analysis further verified effective knockdown at the protein level (Figure 10b,c). Cell proliferation was assessed using CCK‐8 assays at multiple time points. In both SW620 and COLO 201 cells, TIMP1 knockdown resulted in significantly reduced proliferation rates, with both siTIMP1‐1 and siTIMP1‐2 groups showing slower growth compared to controls (Figure 10d,e). We then examined the effect of TIMP1 knockdown on cell migration using wound healing assays. In both cell lines, siTIMP1‐1 and siTIMP1‐2 significantly inhibited wound closure compared to the siNC group, as shown in representative images (Figure 10f,g) and quantitative analysis (Figure 10h), suggesting impaired migratory capacity upon TIMP1 silencing. Transwell migration and invasion assays further demonstrated that TIMP1 depletion significantly reduced both migration and invasion abilities in CRC cells. In COLO 201 and SW620, siTIMP1‐1 and siTIMP1‐2 groups displayed fewer migrated and invaded cells compared to controls, as shown in representative micrographs (Figure 10i,j) and quantified results (Figure 10k,l).

Figure 10Functional validation of TIMP1 knockdown in CRC cell lines. (a–c) Western blot analysis and qRT‐PCR analysis showing TIMP1 protein levels in SW620 and COLO 201 cells after transfection with siNC, siTIMP1‐1, or siTIMP1‐2. (d, e) CCK‐8 assay showing reduced proliferation of (d) SW620 and (e) COLO 201 cells following TIMP1 knockdown at the indicated time points. (f, g) Representative wound healing images of (f) COLO 201 and (g) SW620 cells at 0 and 48 h postscratch. (h) Quantification of wound healing percentage in COLO 201 and SW620 cells. TIMP1 knockdown significantly impaired cell migration in both lines. (i, j) Representative images of Transwell migration and invasion assays in (i) COLO 201 and (j) SW620 cells following TIMP1 knockdown. (k, l) Quantitative analysis of migrated and invaded cells in (k) COLO 201 and (l) SW620. Both migration and invasion capacities were significantly reduced in siTIMP1‐1 and siTIMP1‐2 groups.

**Figure:**
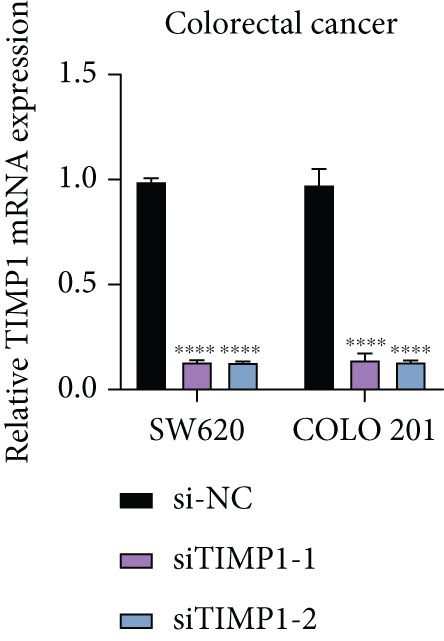
(a)

**Figure:**
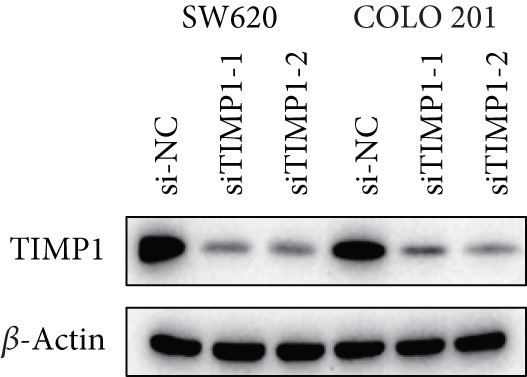
(b)

**Figure:**
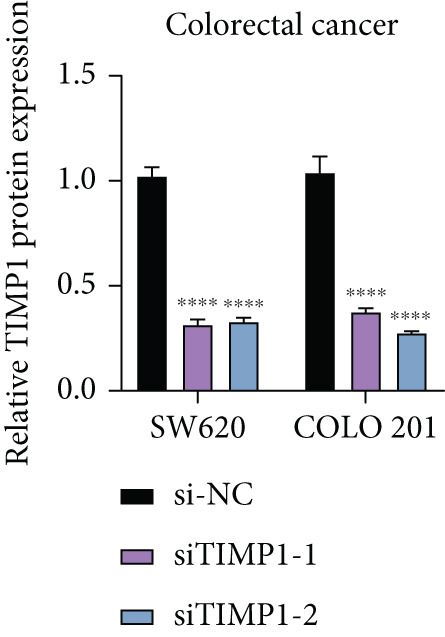
(c)

**Figure:**
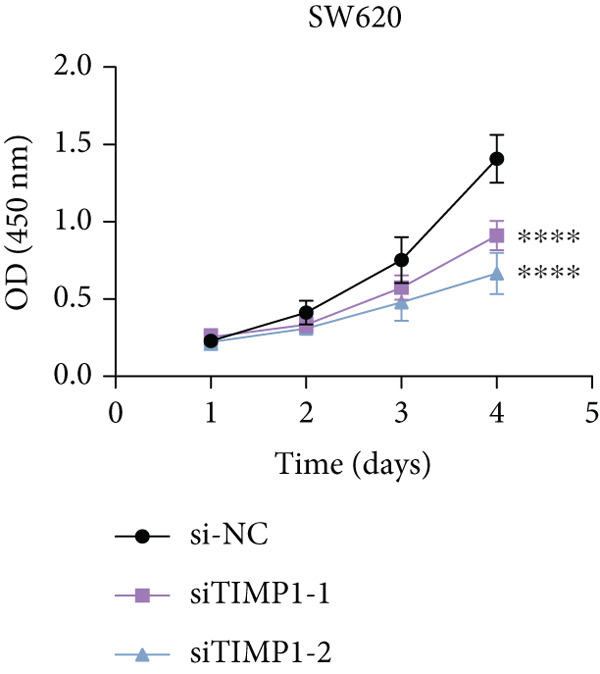
(d)

**Figure:**
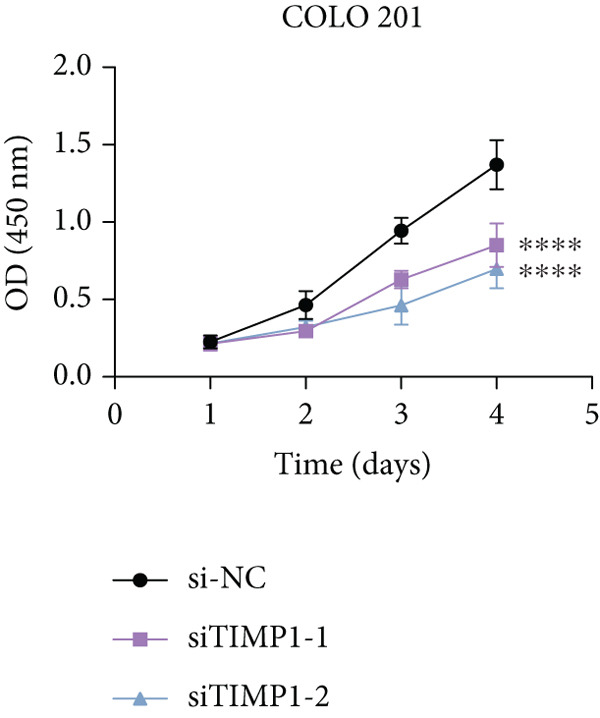
(e)

**Figure:**
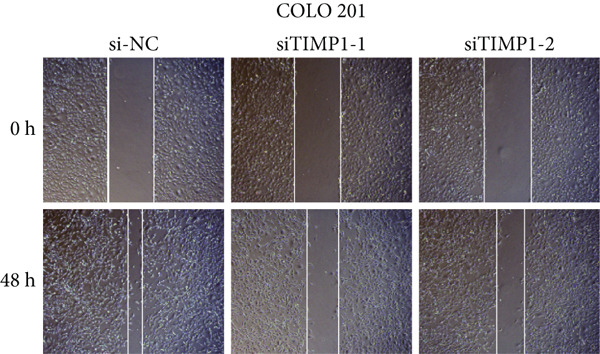
(f)

**Figure:**
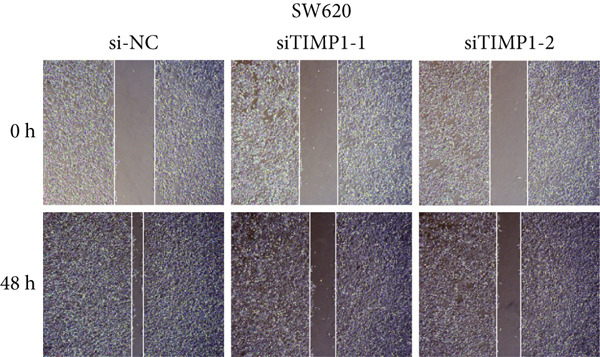
(g)

**Figure:**
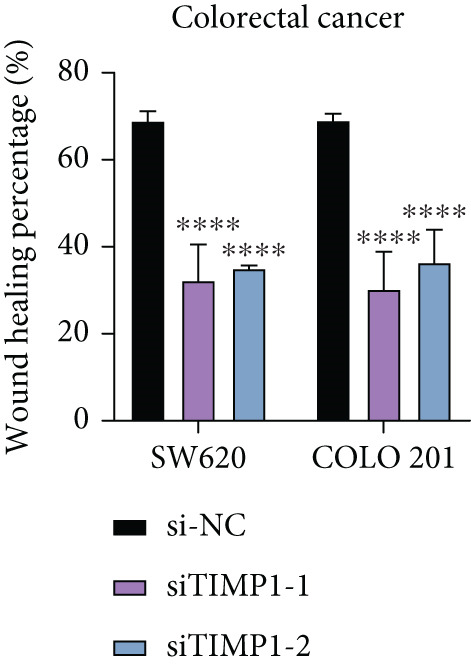
(h)

**Figure:**
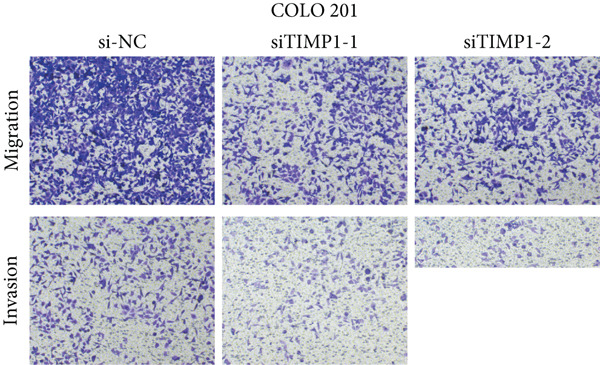
(i)

**Figure:**
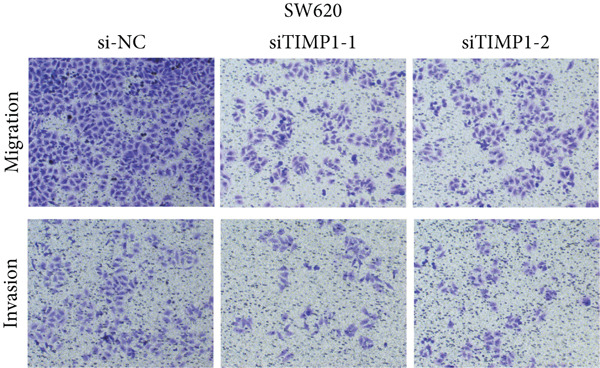
(j)

**Figure:**
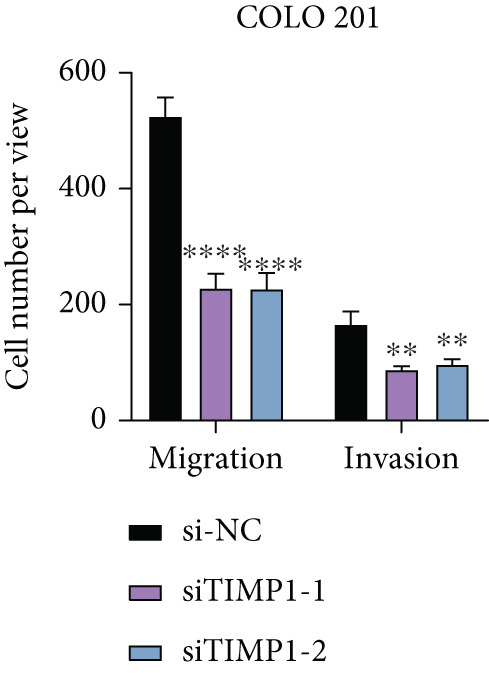
(k)

**Figure:**
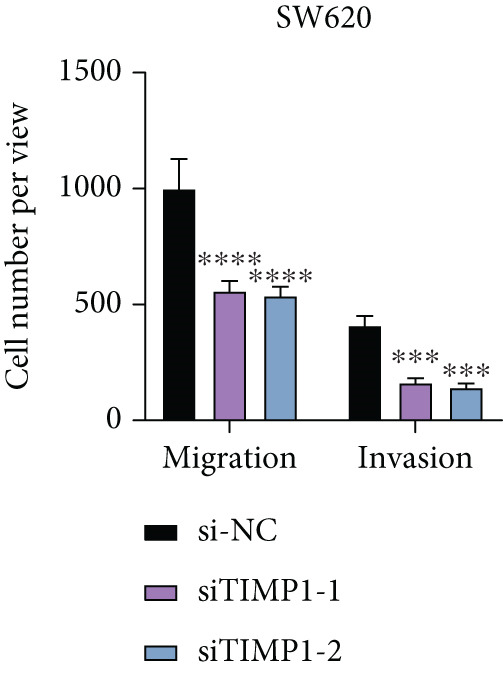
(l)

## 4. Discussion

### 4.1. Biological Functions and Synergistic Effects of Prognostic Genes

The 10 prognostic genes selected in this study have clear biological significance for colon cancer progression and may affect tumor occurrence and development through synergistic effects. For example, TIMP1, as a matrix metalloproteinase inhibitor, can promote tumor invasion and angiogenesis by inhibiting matrix degradation, and its high expression is associated with poor prognosis in colon cancer. CD36 is involved in fatty acid metabolism and can act as a molecule in tumor cell reprogramming, and its abnormal expression can enhance the proliferation and survival ability of tumor cells. CDKN2A is an important tumor suppressor gene, and its deletion or mutation will lead to disordered cell cycle regulation and promote unlimited cell proliferation. The expression patterns of the above genes are significantly different between tumor and adjacent tissues and are closely related to patient prognosis, so they may serve as key regulatory factors in colon cancer progression. In addition, the co‐expression patterns among genes suggest that they may affect tumor progression by synergistically regulating pathways such as cell cycle, metabolic reprogramming, and immune suppression. For example, the positive correlation between CXCL1 and ETS2 may jointly promote the formation of an inflammatory microenvironment, and the negative correlation between PLEC and TUBB2 may mediate the balance between cytoskeleton remodeling and invasion ability. In‐depth exploration of the synergistic mode of the above genes may bring new ideas for targeted therapy of colon cancer.

### 4.2. Clinical Significance of Molecular Typing and Tumor Heterogeneity

The NMF molecular typing constructed using 10 prognostic genes divided colon cancer into 4 subtypes, and each subtype had highly different clinical features, prognosis, and biological functions, further reflecting the high heterogeneity of colon cancer. Among them, C1 had the worst prognosis and was enriched in advanced tumors and immune exclusion types, which may indicate that it has more aggressive biological characteristics and immune escape ability. C3 had the best prognosis, was enriched in early tumors, and was associated with immune inflammation types, indicating that it may have good immune response potential. This typing can not only provide a reference for understanding the biological characteristics of colon cancer but also guide the clinical treatment of colon cancer patients. For example, it may be considered to combine immunotherapy with targeted therapy for C1, while C3 may be more suitable for traditional chemotherapy or immune checkpoint inhibitor therapy. The correlation analysis between molecular typing and clinical features suggests that indicators such as age and tumor stage may be related to the distribution of molecular typing. Therefore, in clinical practice, the combination of molecular typing and clinical features is needed to obtain accurate judgments that are more helpful for prognosis evaluation and treatment selection.

### 4.3. Advantages of Multiomics Integration and Clinical Value of the Model

This study integrated bulk transcriptomics, single‐cell transcriptomics, and spatial transcriptomics data to overcome the limitations of a single technology and analyze the functional and distribution characteristics of prognostic genes from multiple aspects. Bulk data provided the overall gene expression and macro characteristics related to prognosis, single‐cell data provided cell specificity of gene function, and spatial transcriptomics data showed the spatial heterogeneity of gene expression. The combination of the three made the study more comprehensive and reliable. The constructed Enet (*α* = 0.2) prognostic model had better predictive performance in multiple datasets than traditional clinical indicators and published models. This advantage came from the strict gene screening process, the method of integrating multiple algorithms for modeling, and the cross‐validation process integrating multiomics data. The integration of risk scores and clinical indicators in the nomogram model further facilitated clinical application and could provide an intuitive quantitative platform for doctors’ prognosis evaluation. The correlation analysis between the model and TME, immunotherapy response, and drug sensitivity not only verified the biological rationality of the model but also provided more ideas for personalized treatment. For example, high‐risk patients may benefit from immune checkpoint inhibitors and specific chemotherapeutic drugs.

## 5. Limitations and Future Directions

Despite the comprehensive multiomics analysis and robust prognostic modeling, several limitations should be acknowledged. The mechanistic link between IRG1/itaconate and the 10 identified genes remains unclear, and direct experimental evidence is lacking, warranting further functional studies. TIMP1 was the only gene experimentally validated due to its high HR and established oncogenic role, while the contributions of the remaining nine genes remain untested. All datasets were retrospective and publicly available, with overlapping platforms between training and validation cohorts, which may inflate performance metrics and limit immediate clinical applicability. Additionally, potential confounders such as patient heterogeneity and treatment history were not fully accounted for. Future work integrating mechanistic experiments, prospective clinical cohorts, and multicenter validation is needed to confirm the prognostic relevance of these genes and to translate computational predictions into clinically actionable tools.

## 6. Conclusion

In conclusion, this work evaluated the prognostic value of itaconate and Hallmark pathway–related genes in colorectal cancer based on multiomics data, screened 10 important prognostic genes, and constructed an efficient prognostic model to predict patients’ prognosis, immunotherapy response, and drug sensitivity, suggesting that this prognostic model can also play an important role in individualized treatment of colorectal cancer. In addition, this paper analyzed the cell specificity and spatial distribution of prognostic genes through single‐cell and spatial transcriptomics data, providing a more in‐depth and comprehensive understanding of colorectal cancer from the microenvironment level. Overall, itaconate and Hallmark pathway–related genes are related to colon cancer progression, and the prognostic model based on these genes is worthy of promotion in clinical practice.

## Disclosure

All authors reviewed and approved the final version of the manuscript and agree to be accountable for the accuracy and integrity of the work.

## Conflicts of Interest

The authors declare no conflicts of interest.

## Author Contributions

Tingting Zhang and Jianchao Meng contributed to the conceptualization, methodology, formal analysis, software development, visualization, and drafting of the manuscript. Qingyun Wang and Peng Zhang were responsible for data curation and validation. Hui Li, Hailang Wei, and Chen Bai provided resources and contributed to data interpretation and manuscript revision. Denggang Chen, Chen Bai, and Hailang Wei supervised the study and provided project administration. Tingting Zhang and Jianchao Meng contributed equally to this work.

## Funding

No funding was received for this manuscript.

## Data Availability

All datasets utilized or generated during the study are fully available within this article.
